# Methyl jasmonate-induced emission of biogenic volatiles is biphasic in cucumber: a high-resolution analysis of dose dependence

**DOI:** 10.1093/jxb/erx244

**Published:** 2017-08-29

**Authors:** Yifan Jiang, Jiayan Ye, Shuai Li, Ülo Niinemets

**Affiliations:** 1 Institute of Agricultural and Environmental Sciences, Estonian University of Life Sciences, Kreutzwaldi, Tartu, Estonia; 2 College of Art, Changzhou University, Gehu, Changzhou, Jiangsu, China; 3 Estonian Academy of Sciences, Kohtu, Tallinn, Estonia

**Keywords:** Biotic stress, cucumber, dose–response, LOX products, MeJA, proton-transfer reaction time-of-flight mass spectrometer (PTR-TOF-MS), terpenes

## Abstract

Methyl jasmonate (MeJA) is a key airborne elicitor activating jasmonate-dependent signaling pathways, including induction of stress-related volatile emissions, but how the magnitude and timing of these emissions scale with MeJA dose is not known. Treatments with exogenous MeJA concentrations ranging from mild (0.2 mM) to lethal (50 mM) were used to investigate quantitative relationships among MeJA dose and the kinetics and magnitude of volatile release in *Cucumis sativus* by combining high-resolution measurements with a proton-transfer reaction time-of-flight mass spectrometer (PTR-TOF-MS) and GC-MS. The results highlighted biphasic kinetics of elicitation of volatiles. The early phase, peaking in 0.1–1 h after the MeJA treatment, was characterized by emissions of lipoxygenase (LOX) pathway volatiles and methanol. In the subsequent phase, starting in 6–12 h and reaching a maximum in 15–25 h after the treatment, secondary emissions of LOX compounds as well as emissions of monoterpenes and sesquiterpenes were elicited. For both phases, the maximum emission rates and total integrated emissions increased with applied MeJA concentration. Furthermore, the rates of induction and decay, and the duration of emission bursts were positively, and the timing of emission maxima were negatively associated with MeJA dose for LOX compounds and terpenoids, except for the duration of the first LOX burst. These results demonstrate major effects of MeJA dose on the kinetics and magnitude of volatile response, underscoring the importance of biotic stress severity in deciphering the downstream events of biological impacts.

## Teaser

Release of stress volatiles—methanol, lipoxygenase pathway compounds, and monoterpenes and sesquiterpenes—from cucumber leaves upon exposure to methyl jasmonate (MeJA) is biphasic and MeJA dose dependent.

## Introduction

Endogenous levels of jasmonic acid (JA) and its methylated derivative methyl jasmonate (MeJA) are known to increase rapidly in response to herbivore attack or invasion by pathogens, subsequently activating downstream defense responses. Once released into the air, MeJA has been shown to act as a long-distance airborne signal to trigger defense responses in non-impacted parts of the damaged plant or in neighboring plants (Heil and Ton, 2008; Tamogami *et al.*, 2008; Cheong and Choi, 2003). Thus, exogenous application of MeJA has often been used to simulate the impact of a biotic stress on elicitation of jasmonate-dependent defenses (Zhao and Chye, 1999; Heijari *et al.*, 2008; Tamogami *et al.*, 2008; Suh *et al.*, 2013; Shi *et al.*, 2015; Jiang *et al.*, 2016*a*). Among these induced defenses, elicitation of the release of volatile emissions in MeJA-treated leaves is a characteristic MeJA response (Dicke *et al.*, 1999; Kappers *et al.*, 2010; Mäntylä *et al.*, 2014). The chemical classes of plant volatile blends induced by MeJA vary considerably in quantity, quality, and timing, with green leaf volatiles [lipoxygenase (LOX) compounds] and various terpene compounds (mainly monoterpenes and sesquiterpenes) being the typical elicited volatiles (Rodriguez-Saona *et al.*, 2001; Martin *et al.*, 2003; Semiz *et al.*, 2012; Kegge *et al.*, 2013). These emissions occur as the result of both constitutive activity of key stress pathways (e.g. constitutive LOX activities leading to rapid emission of green leaf volatiles; Andreou and Feussner, 2009) and activation of expression of genes responsible for specialized volatile synthesis (e.g. elicitation of expression of terpene synthases leading to emissions of mono- and sesquiterpenes; Martin *et al.*, 2002, 2003; Byun-McKay *et al.*, 2006). Despite the diversity, these induced emissions resemble the emissions induced by herbivores or by physical wounding, and can serve as infochemicals in attracting herbivore enemies or in priming defenses in neighboring plants (Dicke *et al.*, 1999; Heil and Kost, 2006; Heil and Ton, 2008; Kappers *et al.*, 2010), thus underscoring the biological significance of MeJA as a model of chemical signaling.

So far, the majority of studies on the relationships of stress-driven volatile emissions and biotic stress, including herbivore infestation and exogenous MeJA application, have been non-quantitative and have not focused on understanding how much is emitted in response to a certain elicitor dose. The studies have rather mainly looked at the modifications in volatile profiles or at the ecological roles of the volatile induction, for example in plant indirect defenses. However, there is encouraging evidence that the stress-dependent elicitation of volatile emissions is linked to the severity of biotic impacts in a dose-dependent manner (Niinemets *et al.*, 2013). The severity of biotic stress has been modified by varying the degree of wounding (Mithöfer *et al.*, 2005; Portillo-Estrada *et al.*, 2015, 2017) or varying the number of feeding larvae (Copolovici *et al.*, 2011; Yli-Pirilä *et al.*, 2016; Copolovici *et al.*, 2017). However, the definition of the severity of biotic stress, the stress ‘dose’, is still difficult in biotic stress studies because of localized spread and complexity of timing of biotic impacts, especially for multiple biotic impacts occurring at somewhat different times, such as simultaneous herbivore feeding and spread of pathogen infections. Such complex impacts can lead to emission responses that are hard to decipher, complicating construction of mechanistic quantitative stress severity versus emission response models for prediction of signaling among neighboring plants and other organisms (Grote *et al.*, 2013). As exogenous MeJA can be applied in precisely defined doses, it can provide an invaluable model system to simulate dose dependencies of biotic impacts, and start resolving complex biological interactions, at least using volatile responses as a quantitative measure of biotic stress severity.

A dose dependence between the exogenous MeJA concentration and plant volatile response can be expected because treatments with a higher concentration probably result in a greater coverage of potential impact sites in cell wall and cellular membrane surfaces. Furthermore, MeJA exposure has also been related to the downstream components of signaling pathways in cell death regulation (Jonak *et al.*, 2002), and studies using higher concentrations of exogenous MeJA have reported hypersensitive responses including necrosis and/or activation of programmed cell death (PCD) (Popova *et al.*, 2003; Jung, 2004; Zhang and Xing, 2008; Repka *et al.*, 2013) that are expected to result in profound modifications in the total amount and profiles of volatile emission (Beauchamp *et al.*, 2005; Niinemets, 2010). Although the evidence suggests that MeJA activates defense pathways in a dose-dependent manner, to our knowledge, the way in which the MeJA dose alters the timing and magnitude of induced volatile responses has not been studied.

We used cucumber (*Cucumis sativus* L.), known to respond strongly to MeJA (Bouwmeester *et al.*, 1999; Kappers *et al.*, 2010), as the model to investigate the effect of different exogenous MeJA concentrations through early and late phases of MeJA response by combining high-resolution measurements with a proton-transfer reaction time-of-flight mass spectrometer (PTR-TOF-MS) and GC-MS measurements, and kinetic analyses (Table 1). We asked how MeJA dose alters the total amount of volatiles released, how it affects volatile composition, and how it modifies the kinetics of volatile release. We hypothesized that there are MeJA dose-dependent differences in the overall degree of elicitation and compositions of induced volatile emissions. The results of the current study highlight biphasic emission kinetics of volatile emission and strong quantitative relationships between MeJA concentration and the timing and magnitude of early and late emission responses.

**Table 1.** Definition of the traits characterizing the kinetics of volatile compounds released upon methyl jasmonate treatment (see Fig. 2 for representative emission kinetics)

Symbol (unit)	Definition
*D*_M_ (h)	Duration between the first and the second emission maxima
*D*_P1_ (h)	Duration of the first induced emission peak
*D*_P2_ (h)	Duration of the second induced emission peak
*I*_T1_ (nmol m^–2^)	Integral of the first emission peak (Equation 1)
*I*_T2_ (nmol m^–2^)	Integral of the second emission peak
*I*_Tot_ (nmol m^–2^)	Integral of the total induced emissions, *I*_T1_+*I*_T2_
*k*_I1_ (h^–1^)	Rate constant for the initial increase of emissions during the first emission burst (Equation 2)
*k*_D1_ (h^–1^)	Rate constant for the decrease of emissions during the first emission burst (Equation 3)
*k*_I2_ (h^−1^)	Rate constant for the increase of emissions during the second emission burst
*k*_D2_ (h^−1^)	Rate constant for the decrease of emissions during the second emission burst
*t*_M1_ (h)	Time to the first emission maximum since the start of the treatment
*t*_M2_ (h)	Time to the second emission maximum since the start of the treatment
*t*_P1S_ (h)	Start of the first emission burst since the start of the treatment
*t*_P1E_ (h)	End of the first emission burst since the start of the treatment
*t*_P2S_ (h)	Start of the second emission burst since the start of the treatment
*t*_P2E_ (h)	End of the second emission burst since the start of the treatment
*τ*_I1_ (h)	Doubling time for the increase of emissions during the first emission burst
*τ*_D1_ (h)	Half-time for the decrease of emissions during the first emission burst
*τ*_I2_ (h)	Doubling time for the increase of emissions during the second emission burst
*τ*_D2_ (h)	Half-time for the decrease of emissions during the second emission burst
Φ(*t*) (nmol m^−2^ s^−1^)	Emission rate at time *t*
Φ_M1_ (nmol m^−2^ s^−1^)	Maximum emission rate at the first emission peak
Φ_M2_ (nmol m^−2^ s^−1^)	Maximum emission rate at the second emission peak

## Materials and methods

### Plant growth conditions

Cucumber (*Cucumis sativus* cv. Libelle F1, Seston Seemned OÜ, Estonia) seeds were sown in 1 liter plastic pots filled with a mixture (1:1) of sand and commercial potting soil (Biolan Oy, Finland), and cultivated in an environment-controlled plant growth room (Copolovici *et al.*, 2012). In brief, light intensity of 300–400 μmol m^−2^ s^−1^ at the level of plants was provided for 12 h by Philips HPI/T Plus 400 W metal halide lamps (Philips Eesti, Tallinn, Estonia). Air temperature was 24 °C during the day and 20 °C at night, and relative humidity was maintained at 60–70% through the day and night. Plants were watered daily to field capacity, and fertilized every 3 d with a commercial NPK fertilizer (N-P_2_O_5_-K_2_O: 19-5-13). Approximately 3- to 4-week-old, 20–30 cm tall plants with four to five fully expanded leaves were used in the experiments.

### Methyl jasmonate (MeJA) treatments

As studies have used widely different protocols for MeJA application (e.g. Thaler *et al.*, 2002; Loivamäki *et al.*, 2004; Heijari *et al.*, 2005; Liang *et al.*, 2006; Phillips *et al.*, 2007), we tested different methods in preliminary experiments. These tests included different solvents (water with 0.01% Triton X-100 versus 5% ethanol), mode of treatment (spraying versus brushing), treatment location (ventilated hood versus an experimental ventilated flow-through glass chamber; see the details in ‘Dynamic headspace collection of volatiles’). The key selection criteria for MeJA treatment were the repeatability of the treatment in terms of quantitative volatile response and minimization of non-specific effects as assessed by comparing the volatile emissions of non-treated plants and control plants treated with pure solvent identically to the MeJA treatment. Ultimately, application of MeJA (Sigma-Aldrich, St Louis, MO, USA) in 5% aqueous ethanol by spraying in the experimental glass chamber as in several previous studies (Martin *et al.*, 2003; Semiz *et al.*, 2012; Jiang *et al.*, 2016*b*) was selected as the most repeatable application procedure that was associated with minor non-specific effects in control treatments. In fact, volatile emissions in the control treatment (5% ethanol solution) did not significantly differ from non-treated plants.

To obtain the MeJA dose response, the following concentrations were used: 0 (control, 5% ethanol), 0.2, 2, 5, 10, 20, and 50 mM. The selected leaf with an area of ~40 cm^2^ was sealed in the glass gas-exchange cuvette of one of the two gas-exchange systems described below, the baseline measurement of volatile emissions was taken, the cuvette was opened, and 10 ml of MeJA solution was sprayed over the entire leaf surface to obtain a complete and even coating. Immediately after the treatment, the treated leaf was sealed in the gas exchange cuvette again (within ~1 min) and, depending on the system used, volatiles were collected at intervals and analyzed offline by GC-MS or monitored continuously using an online PTR-TOF-MS. Three different plants were used for each MeJA concentration treatment.

### Dynamic headspace collection of volatiles

Volatile collection for GC-MS analysis was carried out with a multichamber open gas-exchange system described in detail by Toome *et al.* (2010) and Copolovici *et al.* (2011) that was also used for MeJA treatments as described above. Each 3 liter glass chamber was operated individually using purified ambient air for the chamber inlet and maintaining an air flow rate of 1 l min^−1^ through the chamber. Turbulent conditions inside the chambers were achieved by a fan installed at the bottom of each individual chamber. The light regime during measurements followed growth light conditions, with light intensity of 200–400 μmol m^−2^ s^−1^ provided for 12 h per day with a Heliospectra LX60 plant growth LED lamp (Heliospectra AB, Sweden). The temperature inside the chambers was between 24 and 26 °C during the light period and 22 °C during the dark period, air humidity was ~60%, and CO_2_ concentration was 380 μmol mol^−1^.

Volatiles in the chamber air were collected onto multibed stainless steel cartridges (10.5 cm length, 4 mm inner diameter; Supelco, Bellefonte, PA, USA) using a constant flow air sample pump (210-1003MTX; SKC Inc., Houston, TX, USA) operated at a rate of 200 ml min^−1^ for 20 min, resulting in quantitative adsorption of volatiles from 4 liters of air. The cartridges were filled with Carbotrap C 20/40 mesh (0.2 g), Carbopack B 40/60 mesh (0.1 g), and Carbotrap X 20/40 (0.1 g) adsorbents (Supelco) for optimal adsorption of volatiles in the C5–C15 range (Kännaste *et al.*, 2014). Before the collection of volatiles, the traps were cleaned by passage of a stream of ultra pure helium at a flow rate of 200 ml min^−1^ at 400 °C for 2 h using a SIM Clean-Cube cartridge thermo-conditioner (Scientific Instruments Manufacturer GmbH, Oberhausen, Germany). After each treatment, the chamber and tubing were flushed with a stream of ozone (~1000 ppm) to eliminate the volatiles adsorbed by the chamber and tubes that could contaminate the measurement system (mainly MeJA, and to a low degree stress-induced volatiles; Niinemets *et al.* 2011).

**Fig. 1.**
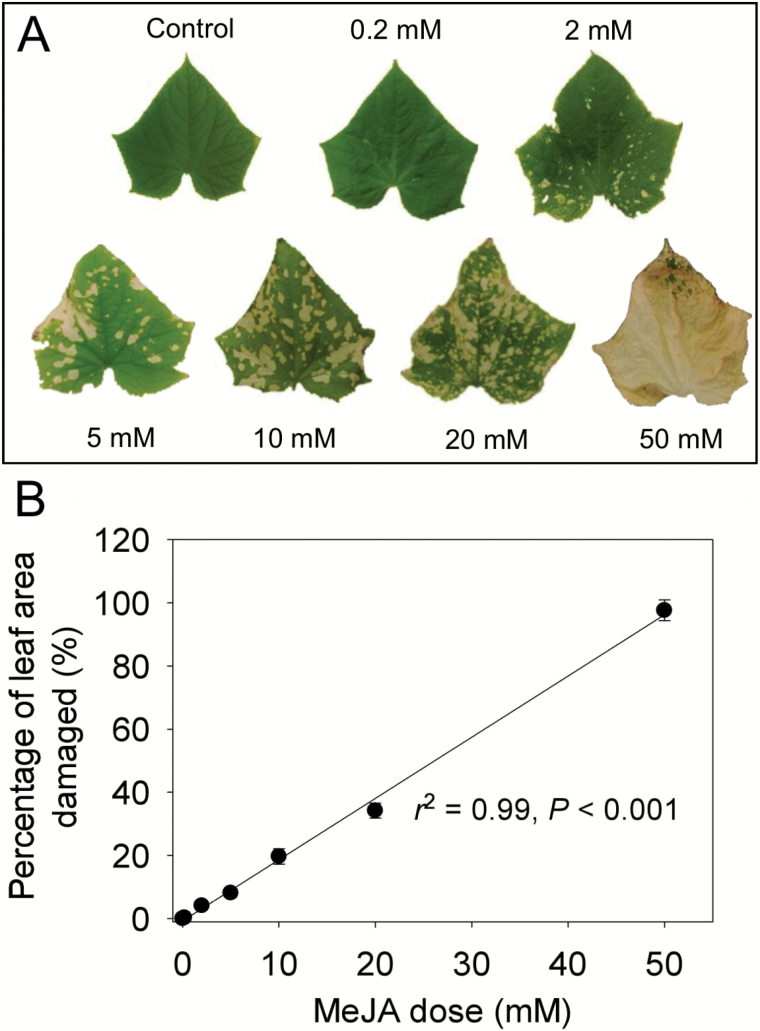
Characteristic images of MeJA-treated cucumber (*Cucumis sativus*) leaves taken 36–48 h after treatment with MeJA concentrations of 0 (control), 0.2, 2, 5, 10, 20, and 50 mM (A), and corresponding relationship between the percentage of damage and applied MeJA concentration (B). The data in (B) were fitted by a linear regression (*y*=1.94*x*–0.71; *r*^2^=0.99, *P*<0.001).

The volatile samples were collected before leaf treatment with MeJA and 20 min, 2, 10, and 24 h after treatment with MeJA. Blank empty chamber measurements were taken before and after the experiment. During and after the experiment, additional blank samples were taken from the adjacent empty chamber operated under identical conditions, and the baseline during the experiment was adjusted when needed using the blanks from the experimental chamber and empty chamber at the end of the experiment (the difference between the two blanks was small, indicating that the system memory effect was minor). After 36–48 h, the experiment was finished, and the treated leaf was removed, scanned, and its area was estimated with the UTHSCSA ImageTool 2.0 (Dental Diagnostic Science, The University of Texas Health Science Center, San Antonio, TX, USA). These leaf images were further used to assess the quantitative degree of damage (Fig. 1A).

### Gas-chromatographic analysis of volatiles

Adsorbent cartridges were analyzed according to the method of Kännaste et al. (2014) using a combined Shimadzu TD20 automated cartridge desorber and a Shimadzu 2010 Plus gas chromatograph with mass spectrometric detector (GC-MS; Shimadzu Corporation, Kyoto, Japan). A C8–C20 hydrocarbon standard (Sigma-Aldrich) was used to obtain the retention indices as in Pazouki *et al.* (2015) and in Jiang *et al.* (2016*b*) (Table 2). Compounds were identified using the NIST spectral library, the spectral and retention indices library of Adams (1995), and a custom-made library of retention times and mass spectra of commercially available mono- and sesquiterpene standards (GC purity, Sigma-Aldrich). The authentic standards were also used for GC-MS calibration as described in detail in Kännaste *et al.* (2014). The background (blank) concentrations of individual volatiles in the empty chamber were subtracted from the plant samples, and the emission rates were calculated according to the equations of Niinemets *et al.* (2011).

**Table 2.** Average (± SE) emission rates (pmol m^-2^ s^-1^) of individual volatile compounds after treatment of MeJA with different doses over the time-course of 24 h identified by GC-MS

Volatile Compounds	Retention index	20 min					2 h						10 h					24 h				
		Control	2 mM	5 mM	10 mM	20 mM	Control	2 mM	5 mM	10 mM	20 mM	Control	2 mM	5 mM	10 mM	20 mM	Control	2 mM	5 mM	10 mM	20 mM
Lipoxygenase pathway products and saturated aldehydes																						
(*E*)-3-Hexenal	802	120 ± 24a	167 ± 37a	608 ± 87ab	663 ± 87ab	860 ± 140b	80 ± 17	71 ± 11	110 ± 24	180 ± 24	130 ± 20	76 ± 20a	46.5 ± 6.8a	298 ± 62ab	820 ± 130b	720 ± 150b	63 ± 14	81 ± 12	123 ± 14	161 ± 22	169 ± 19
(*Z*)-3-Hexen-1-ol	863	ND^a^	ND	130 ± 17a	342 ± 68ab	1000 ± 140b	ND	ND	4.7 ± 1.9	5.7 ± 0.7	10.1 ± 2.4	ND	18.9 ± 2.1a	64 ± 12ab	304 ± 43b	252 ± 23b	ND	ND	ND	ND	ND
Heptanal	899	ND	ND	101 ± 18	182 ± 39	152 ± 22	ND	ND	ND	115 ± 12	76 ± 17	ND	ND	102 ± 11	223 ± 37	233 ± 29	ND	ND	20.2 ± 3.3	27.9 ± 3.0	21.3 ± 1.9
Octanal	1074	105 ± 19	183 ± 32	110 ± 17	201 ± 30	161 ± 21	177 ± 24	223 ± 22	94 ± 22	219 ± 16	167 ± 24	101 ± 17	131 ± 18	167 ± 24	42.8 ± 9.9	98 ± 16	66 ± 14	137 ± 17	144 ± 15	109 ± 18	140 ± 19
*(Z*)-3-Hexen-1-yl acetate	1008	ND	ND	2160 ± 290	1930 ± 210	3270 ± 810	ND	ND	ND	ND	ND	ND	ND	ND	ND	ND	ND	ND	ND	ND	ND
2-Ethyl-1-hexanol	1046	130 ± 31	229 ± 63	137 ± 18	249 ± 37	201 ± 28	222 ± 27	278 ± 32	118 ± 22	273 ± 29	208 ± 27	127 ± 19	163 ± 23	208 ± 26	53.9 ± 9.3	122 ± 23	83 ± 16	172 ± 11	180 ± 17	136 ± 23	176 ± 24
Nonanal	1098	502 ± 74	868 ± 99	521 ± 99	950 ± 170	763 ± 124	843 ± 112	1050 ± 140	446 ± 81	1042 ± 81	790 ± 140	484 ± 87	620 ± 110	794 ± 93	204 ± 29	465 ± 99	316 ± 63	650 ± 120	682 ± 99	515 ± 87	660 ± 120
Decanal	1204	561 ± 99	977 ± 81	590 ± 120	1060 ± 150	857 ± 112	945 ± 87	1180 ± 170	505 ± 93	1166 ± 93	890 ± 120	542 ± 74	699 ± 81	888 ± 81	229 ± 31	523 ± 94	353 ± 62	731 ± 93	769 ± 87	580 ± 93	750 ± 81
Monoterpenes																						
α-Pinene	932	76 ± 11	50 ± 11	81 ± 12	62.6 ± 9.9	86.2 ± 9.9	78 ± 11	57.0 ± 7.4	41.5 ± 9.3	58.9 ± 8.1	65.7 ± 8.7	64.5 ± 9.3	100 ± 11	99.2 ± 9.3	182 ± 38	246 ± 34	107 ± 14	52.7 ± 7.4	39.1 ± 4.3	36.0 ± 5.6	35.3 ± 4.3
Camphene	949	1.4 ± 0.4	1.4 ± 0.6	1.0 ± 0.4	2.0 ± 0.3	2.4 ± 0.6	1.9 ± 0.3	1.9 ± 0.5	1.4 ± 0.3	3.6 ± 0.6	2.9 ± 0.4	2.2 ± 0.4	3.0 ± 0.3	5.1 ± 0.9	9.9 ± 2.1	15.5 ± 4.3	0.62 ± 0.09a	2.0 ± 0.4ab	3.41 ± 0.682b	6.51 ± 0.87bc	8.7 ± 1.1c
β-Pinene	980	3.3 ± 1.0	4.3 ± 1.2	3.6 ± 0.7	2.9 ± 0.6	3.3 ± 1.0	2.2 ± 0.6	3.5 ± 0.9	4.7 ± 1.1	6.4 ± 0.9	6.1 ± 0.9	2.6 ± 0.4	2.9 ± 0.6	5.8 ± 1.1	5.8 ± 1.8	8.1 ± 1.6	0.66 ± 0.06	2.2 ± 0.4	1.8 ± 0.3	3.2 ± 0.6	2.9 ± 0.4
Δ^3^-Carene	1011	36.6 ± 8.9	31.6 ± 8.7	55.8 ± 9.9	65.7 ± 8.7	71 ± 19	39.1 ± 5.6	64.5 ± 7.4	31.4 ± 5.6	26.0 ± 6.2	55.2 ± 6.8	43.4 ± 5.0a	55.8 ± 8.7a	133 ± 19b	311 ± 50bc	477.4 ± 68c	53.3 ± 8.1	40.3 ± 6.2	29.1 ± 3.1	67.6 ± 8.1	57.0 ± 9.3
Limonene	1029	ND	ND	ND	ND	ND	ND	ND	ND	ND	ND	ND	ND	254 ± 37	428 ± 50	378 ± 50	ND	ND	42.0 ± 8.6	43.2 ± 8.4	99 ± 25
Linalool	1098	ND	ND	ND	ND	ND	ND	ND	ND	ND	ND	ND	ND	7.0 ± 1.0	22.2 ± 2.5	18.0 ± 3.7	ND	ND	ND	ND	ND
Sesquiterpenes																						
α-Cedrene	1409	ND	ND	ND	ND	ND	ND	ND	ND	ND	ND	ND	ND	ND	18.3 ± 3.0	30.4 ± 4.3	ND	ND	ND	9.3 ± 3.1	ND
β-Caryophyllene	1428	ND	ND	ND	ND	ND	ND	ND	ND	ND	ND	ND	ND	ND	4.6 ± 1.1	9.4 ± 2.2	ND	ND	ND	ND	ND
β-Farnesene	1455	ND	ND	ND	ND	ND	ND	ND	ND	ND	ND	ND	7.1 ± 1.9	4.8 ± 0.7	8.7 ± 2.2	13.4 ± 3.0	ND	ND	ND	0.7 ± 0.3	ND
Geranylgeranyl diphosphate (GGDP) pathway derived volatiles^b^																						
6-Methyl-5-hepten-2-one	985	158 ± 29a	366 ± 56ab	185.4 ± 29a	513 ± 58ab	1810 ± 320b	99 ± 17a	252 ± 32ab	231 ± 56ab	455 ± 56ab	1100 ± 170b	152 ± 19	179 ± 25	145 ± 19	199 ± 32	114 ± 12	15.2 ± 2.8	17.9 ± 2.8	214.5 ± 2.5	19.9 ± 2.2	70.1 ± 6.8
Geranylacetone	1453	34.2 ± 1.4	59 ± 13	36.0 ± 5.6	65 ± 11	52.1 ± 5.0	57.7 ± 6.2	73 ± 12	31.4 ± 9.3	71.3 ± 8.1	53.9 ± 8.7	32.9 ± 7.4	42.2 ± 9.3	53.9 ± 7.4	14.0 ± 2.5	31.6 ± 5.0	21.5 ± 2.5	44.6 ± 8.7	46.5 ± 8.7	35.3 ± 3.1	45.9 ± 5.0
Benzenoids																						
Benzaldehyde	967	180 ± 37a	126 ± 19a	236 ± 62a	550 ± 87ab	890 ± 110b	148 ± 18	158 ± 22	167 ± 37	107.9 ± 7.4	137 ± 19	32.9 ± 7.4a	118 ± 12a	210 ± 25ab	515 ± 74ab	869 ± 99b	484 ± 87	205 ± 22	156 ± 19	113 ± 16	68.8 ± 4.3

Three replicate treatments at each MeJA application concentration were carried out. Means among treatments and sampling times were compared by ANOVA followed by post-hoc Tukey’s tests, and statistically significant differences are denoted by different lowercase letters. For compounds without labels, the emission rates did not differ significantly among treatments at different sampling events.

The pathways leading to saturated aliphatic aldehydes are not yet fully resolved, although these emissions are also up-regulated upon abiotic and biotic stresses similarly to lipoxygenase volatiles (Wildt et al., 2003; Hu et al., 2009)

The data of the 50 mM treatment are not shown in the table because this lethal dose caused a rapid necrosis in 1 h and no volatile emission was detected then from the leaves, implying the loss of the biological activity.

^*a*^ ND, below the detection limit of ~0.1 pmol m^−2^ s^−1^

^*b*^ Carotenoid breakdown products including geranyl acetone (Gao *et al.*, 2008; Arimura *et al.*, 2009; Kask *et al.*, 2016).

### Online monitoring of the kinetics of volatile release

A high-resolution PTR-TOF-MS (TOF8000, Ionicon Analytik, Innsbruck, Austria) was used to track the volatile release in real time. The PTR-TOF-MS system was connected to a custom-designed two-channel gas-exchange system described in detail by Copolovici and Niinemets (2010). The measurement cuvette (1.2 l) consisted of a stainless steel bottom and a double-walled glass upper part specially designed for volatile compound measurements. A thermostat was used to control the temperature of water circulating between the glass chamber walls, and the chamber air temperature was within ±0.2 ºC of the chamber wall temperature. Four wide-beam halogen lamps (Osram GmbH, Germany) were used for chamber illumination. Ambient air that was purified through the passage of a charcoal filter and an ozone trap, and humidified to the desired water vapor pressure was used (Copolovici and Niinemets, 2010). The flow rate through the system was 1.6 l min^−1^, and the sample air was drawn into the PTR-TOF-MS drift tube at a flow rate of 100 ml min^−1^. Background volatile concentrations in the ambient air pumped into the chamber were assessed in the reference mode, and volatile concentrations in the outgoing air stream were measured in the sample mode. In addition, measurements with the empty chamber were also conducted before and after plant measurements. In these experiments, chamber temperature was maintained at 25 °C, light intensity at the leaf surface at 500 μmol m^−2^ s^−1^, chamber air humidity between 50% and 60% (vapor pressure deficit of 1.2–1.6 kPa), and CO_2_ concentration between 370 μmol mol^−1^ and 400 μmol mol^−1^. After enclosure of the MeJA-treated leaf, all measurements were immediately started.

The operation of the PTR-TOF-MS, system calibration, and compound detection followed the protocol of Portillo-Estrada *et al.* (2015). In brief, the drift tube conditions were 2.3 mbar, 600 V, and 60 °C, the measurements were carried out continuously through the mass to charge ratio (*m*/*z*) range of 0–316, and data for 31 250 spectra s^–1^ were averaged. The raw PTR-TOF-MS data were post-processed with the PTR-MS Viewer 3.0.0.99 (Tofwerk AG, Switzerland), and relevant *m*/*z* peaks were integrated as explained in Portillo-Estrada *et al.* (2015). The time resolution used in this study is 10 s (the averaged data were recorded every 10 s). Methanol was detected as the protonated parent ion with an *m*/*z* of 33, while the total emission of volatiles produced within the octadecanoid pathway (LOX products) was taken as the sum of individual ion masses (*m*/*z*) of 57 [*m*_57_, (*E*)-2-hexenal (frag)], 81 [*m*_81,_ (*Z*)-3-hexenal+(*E*)-3-hexenal (frag) after correction for the share of the fraction originating from monoterpenes; see below], 83 [*m*_83_, hexenol+hexanal (frag)], 85 [*m*_85_, hexanol (frag)], 103 [*m*_103_, hexanol (main)], 99 [*m*_99_, (*Z*)-3-hexenal+(*E*)-3-hexenal (main)], and 101 [*m*_101_, (*Z*)-3-hexenol+(*E*)-3-hexenol+(*E*)-2-hexenol+hexanal (main)]) (Copolovici and Niinemets, 2010; Portillo-Estrada *et al.*, 2015). Total monoterpene emission was characterized by the parent ion with an *m*/*z* of 137 (*m*_137_), and total sesquiterpene emission by the parent ion with *m*/*z* 205 (*m*_205_). As even under the soft ionization conditions of the operation of PTR-TOF-MS used here, monoterpenes can partly fragment, with the fragment spectrum dominated by the mass fragments with *m/z* 67, 81, and 95 (Copolovici *et al.*, 2005; Tani *et al.*, 2003), use of parent ions somewhat underestimates the emissions of monoterpenes. Based on simultaneous GC-MS measurements, for total monoterpenes, we divided the concentration of the parent ion by *m/z* 137 by 0.46 to obtain total monoterpene emission. To consider the interference of the mass fragment with *m/z* 81 coming from monoterpene fragmentation with the detection of the LOX compound 3-hexenal, fragmentation of which also produces the identical mass fragment, we separately analyzed the PTR-TOF-MS fragmentation spectra of (*Z*)-3-hexenal and all emitted terpenoids, and developed appropriate equations to assess the share of *m/z* 81 from monoterpenes and 3-hexenal based on the concentration of corresponding parent ions.

**Fig. 2.**
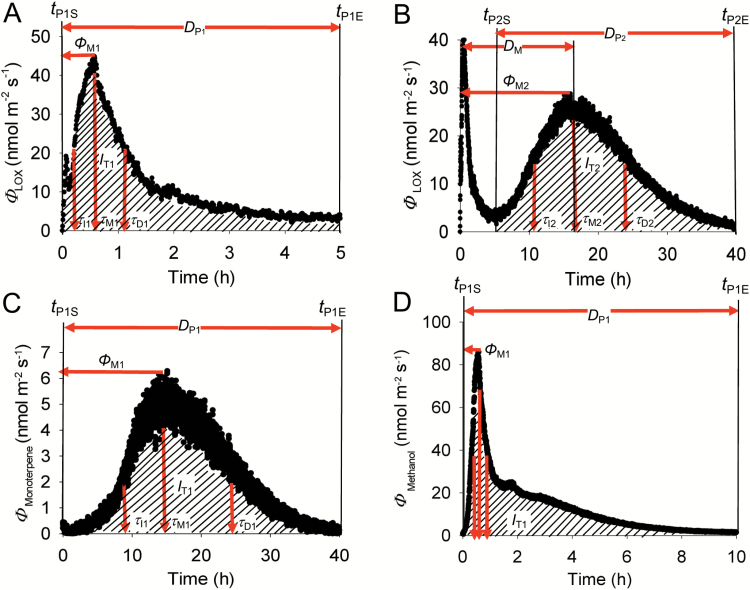
Representative time-courses of methyl jasmonate (MeJA)-induced emissions from a *C. sativus* leaf, and definition of key variables characterizing the induction response (see Table 1 for further specifics). Volatile emissions were induced by application of 20 mM MeJA that elicited an early lipoxygenase pathway volatile (LOX) burst (A), and a second late LOX emission burst (B) with concomitant monoterpene emission burst (C) and an early slowly decaying methanol emission burst (D). Shaded areas in all panels indicate integrated emissions corresponding to individual emission bursts (*I*_T1_ for the first and *I*_T2_ for the second burst, Equation 1), Φ _T1_ and Φ _T2_ correspond to the emission maxima for the two bursts, and *t*_M1_ and *t*_M2_ denote the corresponding times from the start of the treatment. Different τ-s stand for the initial doubling and decay times for the two bursts (Table 1). In (D), the red vertical arrows show the positions of *τ *_I1_, *t*_M1_, and *τ *_D1_ as in the other panels. After the leaf treatment with MeJA, the release of emissions was continuously monitored with a proton-transfer reaction time-of-flight mass spectrometer (PTR-TOF-MS). The time kinetics of sesquiterpene emission is analogous to monoterpene emissions and is therefore not presented.

The emission rates per unit leaf area were calculated according to Niinemets *et al.* (2011) considering both the incoming air measurements taken frequently during the experiments and empty chamber measurements before plant enclosure (typically only differing slightly from the incoming air concentrations). PTR-TOF-MS measurements were continued until the elicited emissions reached the background level, usually between 36 h and 48 h after the treatment (Fig. 2).

### Quantitative characterization of elicitation of volatile emissions by MeJA treatment

Time-courses of emissions induced by MeJA treatment were either biphasic with two maxima (LOX products; Fig. 2A, B) or monophasic (monoterpenes, sesquiterpenes, and methanol; Fig. 2C, D). The emission time-courses were used to derive the key quantitative emission characteristics (Fig. 2; Table 1), including the emission rates at the two emission maxima (Φ_M1_ and Φ_M2_), the corresponding times for the emission maxima (*t*_M1_ and *t*_M2_), the durations of the two emission peaks (*D*_P1_ and *D*_P2_), the duration between the emission maxima (*D*_M_), and the total volatile emissions corresponding to both emission bursts (*I*_T1_ and *I*_T2_). For the first emission burst, the total integrated emission was calculated as:

$$I_{\text{T}1}=\int_{t_{\text{P1S}}}^{t_{\text{P1E}}}\Phi (t)\text{d}t,$$ (1)

where Φ(*t*) is the emission rate at time *t*, *t*_P1S_ is the start, and *t*_P1E_ is the end of the first induced emission release. In practice, numerical integration according to the trapezoidal rule was used and the infinitesimal time period d*t* was replaced by the measurement period Δ*t* of 10 s. The integrated emission corresponding to the second emission burst was calculated analogously.

The emission kinetics of different volatiles followed a similar pattern characterized by an initial exponential increase, then by slowing down of the rate of increase until the emissions reached the maximum value, followed by a non-linear decay to the baseline emission (Fig. 2). We fitted the initial parts of the increase and decay of the emissions corresponding to the first and the second emission burst with simple first-order exponential models. For the first peak,

$$\Phi (t)=\;\Phi \text{(}t_{\text{P1S}}{\text{)e}}^{k_{\text{I1}}t},$$ (2)

for the increasing part, and

$$\Phi (t)=\;\Phi \text{(}t_{\text{M1}}{\text{)e}}^{-k_{\text{D1}}t},$$ (3)

for the decreasing part. Here, *k*_I1_ is the rate constant for the increase and *k*_D1_ is the rate constant for the decrease of emission of the given compound. If present, the rate constants for the increase and decrease for the second peak (*k*_I1_ and *k*_D2_) were calculated analogously. From the rate constants, corresponding doubling times [e.g. for the first emission burst, τ_I1_=ln(2)/*k*_I1_] and decay half-times [τ_D1_=ln(2)/*k*_D1_] for both emissions bursts were also calculated.

### Data analyses

All experiments were replicated three times with different plants, and all data shown are averages ±SE. Effects of MeJA dose were studied by linear or non-linear regressions depending on the shape of the response. Emission rates of volatiles elicited by different MeJA concentrations at fixed time points estimated by GC-MS were compared by ANOVA followed by post-hoc Tukey’s test. The analyses were conducted with SAS (Version 8.02; SAS Institute, Cary, NC, USA) and all statistical effects are considered significant at *P*<0.05.

## Results

### Methyl jasmonate treatments in leaves of *Cucumis sativus*: general patterns and short- and long-term emission responses

Control plants were weak emitters of monoterpenes α-pinene, camphene, β-pinene, and Δ^3^-carene, several longer aliphatic aldehydes C7–C10, some characteristic C6 lipoxygenase pathway volatiles (LOX), and benzaldehyde (Table 2). MeJA treatment resulted in rapid elevation of C6 and derivative LOX products, (*Z*)-3-hexen-1-ol, 3-hexenyl acetate, (*Z*)-3-hexen-1-ol, and *n*-hexanal, detectable with GC-MS already at 20 min after treatment. Moreover, 6-methyl-5-hepten-2-one and heptanal could also be induced significantly by higher concentrations of MeJA (10 mM and 20 mM). Emissions of C6-LOX compounds in the treated plants were strongly reduced at the second sampling event at 2 h, and the emissions increased again at 10 h, especially C6-LOX compounds and derivatives (Table 2). However, 3-hexenyl acetate was not detectable in any of the MeJA dose treatments after the initial elevation at 20 min. In 24 h after the treatment, (*Z*)-3-hexen-1-ol could not be identified in any of the MeJA dose treatments, and the emission rate of *n*-hexanal decreased dramatically compared with the emission rate at 10 h. However, nonanal and decanal emissions remained at a moderately high level throughout the treatments (Table 2).

In contrast to aliphatic aldehydes and LOX, no enhancement of monoterpene emissions and no sesquiterpene emissions in MeJA-treated leaves were observed at the first two measurement events (Table 2). However, monoterpene emissions, especially limonene and Δ^3^-carene emissions, were enhanced in MeJA-treated leaves at 10 h and, at this sampling event, emissions of the monoterpene linalool, and sesquiterpenes β-farnesene, α-cedrene, and β-caryophyllene were identified (Table 2). At 24 h after the treatment, terpenoid emissions had mostly reached the pre-treatment level (Table 2).

In 36–48 h after the exposure to MeJA, all treated leaves except those from the 0.2 mM treatment exhibited a certain degree of damage ranging from chlorotic spots in the 2 mM treatment to major chlorotic areas in 5–20 mM treatments, and at the lethal concentration of 50 mM, the damage was spread over the entire leaf area (Fig. 1A). The damaged leaf area was linearly correlated with the concentration of MeJA applied (Fig. 1B).

High time resolution measurements of kinetics of elicitation of volatile emissions in MeJA-treated leaves by PTR-TOF-MS broadly confirmed the timing of release of different compounds highlighted by GC-MS analyses (Fig. 2). PTR-TOF-MS measurements also confirmed the occurrence of two emission bursts for LOX, a fast burst elicited immediately after MeJA treatment and reaching a maximum in ~0.2–1 h, and a slower burst elicited in 6–10 h after MeJA treatment and reaching a maximum in ~16–20 h (Fig. 2A, B). In the case of mono- (*m*/*z* 137) and sesquiterpenes (*m*/*z* 205), only one slower burst was observed (Fig. 2C). This slower burst started in 2–6 h after MeJA treatment and reached a maximum in ~15–25 h (Fig. 2C). Apart from the compounds detected by GC-MS, PTR-TOF-MS measurements demonstrated a major burst of methanol emission (Fig. 2D). Methanol (*m*/*z* 33) emissions started almost immediately after MeJA treatment, and reached a maximum ~0.5–1.5 h after the treatment (Fig. 2D).

The MeJA threshold concentration for elicitation of both the rapid and the slow LOX emission bursts and the methanol emission burst was 2 mM, while for monoterpenes the threshold concentration was 0.2 mM and for sesquiterpenes 2 mM. The slower emission burst for LOX and the emission bursts for monoterpenes and sesquiterpenes were absent for leaves treated with the highest (lethal, Fig. 1A) MeJA concentration of 50 mM.

### MeJA elicits volatile emissions in a dose-dependent manner

**Fig. 3.**
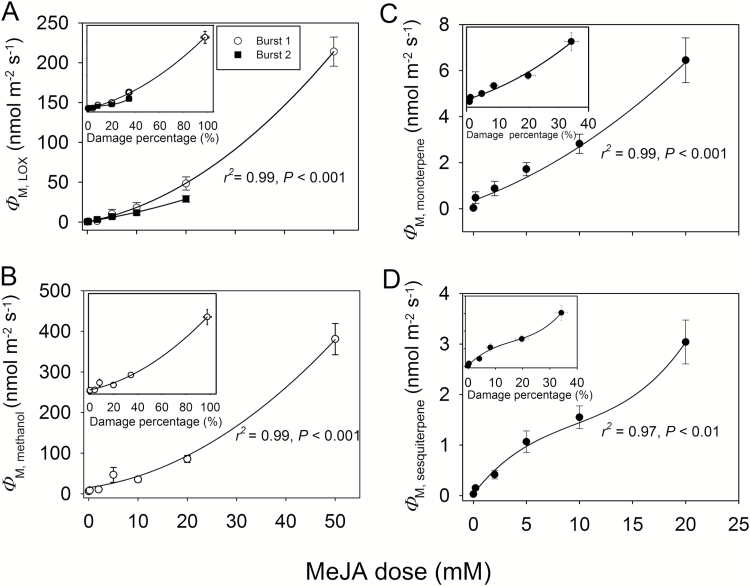
Maximum emission rates of LOX (Φ _M,LOX_, A), methanol (Φ _M,methanol_, B), monoterpenes (Φ _M,monoterpene_, C), and sesquiterpenes (* Φ *_M,sesquiterpene_, D) as dependent on the applied MeJA dose and corresponding correlations with the degree of damage at the end of the experiment (insets) in leaves of *C. sativus* (see Fig. 2 for sample-induced emission kinetics). MeJA treatments as in Fig. 1. Treatment with 50 mM MeJA was lethal (Fig. 1) and, thus, the second LOX emission burst and monoterpene and sesquiterpene emission bursts were absent at this concentration. Data were fitted by second-order polynomial regressions, except that sigmoidal regressions were used for (D). For the first LOX burst in (A), *y*=0.062*x*^2^+1.19*x*+0.46 (main panel) and *y*=0.013*x*^2^+0.89*x*–0.3 (inset). For the second LOX burst in (A), *y*=0.0335*x*^2^+0.94*x*+0.46 (main panel) and *y*=0.013*x*^2^+0.37*x*+1.05 (inset). For methanol in (B), *y*=0.11*x*^2^+1.65*x*+12.0 (main) and *y*=0.026*x*^2^+1.28*x*+10.9 (inset), for monoterpenes in (C), *y*=0.0037*x*^2^+0.23*x*+0.30 (main) and *y*=0.0023*x*^2^+0.098*x*+0.36 (inset), and for sesquiterpenes in (D), *y*=3.26/[1+e^(9.80−*x*)/4.02^] (main) and *y*=3.72/[1+e ^(21.5−*x*)/8.72^]. For the relationships in (A–C), *r*^2^=0.99, *P*<0.001, except for the second LOX burst versus damage percentage in (A) where *r*^2^=0.98, *P*<0.01. In (D), *r*^2^=0.97, *P*<0.01 for the main panel and *r*^2^=0.96, *P*<0.01 for the inset. Three replicate treatments at each MeJA application concentration were carried out.

**Fig. 4.**
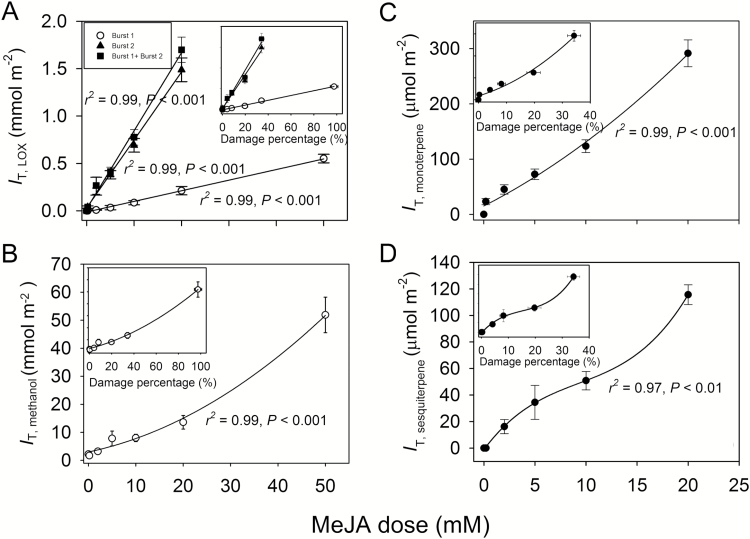
Integrated emissions (*I*_T_, Equation 1) of lipoxygenase pathway volatiles (*I*_T,LOX_, A), methanol (*I*_T,methanol_, B), monoterpenes (*I*_T,monoterpene_, C), and sesquiterpenes (*I*_T,sesquiterpene_, D) in relation to the applied MeJA concentration and corresponding relationships with the degree of damage (insets) in leaves of *C. sativus* (see Fig. 2 for the sample kinetics of the emissions). In (A), the data for the first and the second emission burst of LOX and the sum of the two are separately shown and fitted by linear regressions (for the main panel, *y*=0.0112*x*–0.0124 for the first and *y*=0.0716*x*+0.0322 for the second LOX burst; for the inset, *y*=0.00576*x*–0.0080 for the first and *y*=0.0409*x*+0.023 for the second LOX burst). For *I*_T,methanol_ in (B) and for *I*_T,monoterpene_ in (C), the data were fitted by second-order polynomial regressions [for the main panels, *y*=0.0093*x*^2^+0.99*x*+1.62 for (B) and *y*=0.22*x*^2^+9.43*x*+15.2 for (C); and for the insets, *y*=0.0026*x*^2^+0.25*x*+2.66 for (B) and *y*=0.12*x*^2^+3.85*x*+17.8 for (C)]. For *I*_T,sesquiterpene_ in (D), the data were fitted by sigmoidal regressions {*y*=127/[1+e^(11.3−*x*)/4.20^] for the main panel and *y*=161/[1+e^(25.8−*x*)/9.16^] for the inset}. MeJA treatment is as in Fig. 1. For all relationships in (A) and (B) and for the main panel of (C), *r*^2^=0.99, *P*<0.001. For the inset of (C), *r*^2^=0.98, *P*<0.01 and for the main panel and the inset of (D), *r*^2^=0.96, *P*<0.01. Three replicate treatments at each MeJA application concentration were carried out.

The maximum emission rate of MeJA-elicited emissions increased with increasing MeJA concentration for both the faster (Fig. 3A, B) and the slower (Fig. 3A, C, D) MeJA response. The dependencies of the maximum emission rates of volatiles on MeJA concentration were somewhat non-linear, implying that the increases of the maximum emission rates were greater at greater MeJA treatment concentration, especially between the concentrations of 10 mM and 20 mM and 20 mM and 50 mM (Fig. 4). The emission maxima scaled positively with the degree or leaf damage (insets in Fig. 3). MeJA dose dependencies of emission rates of LOX in the early response phase and of LOX and mono- and sesquiterpenes in the late phase were also evident in GC-MS data. However, due to the lower time resolution, the variability in these responses was greater (Table 2).

**Fig. 5.**
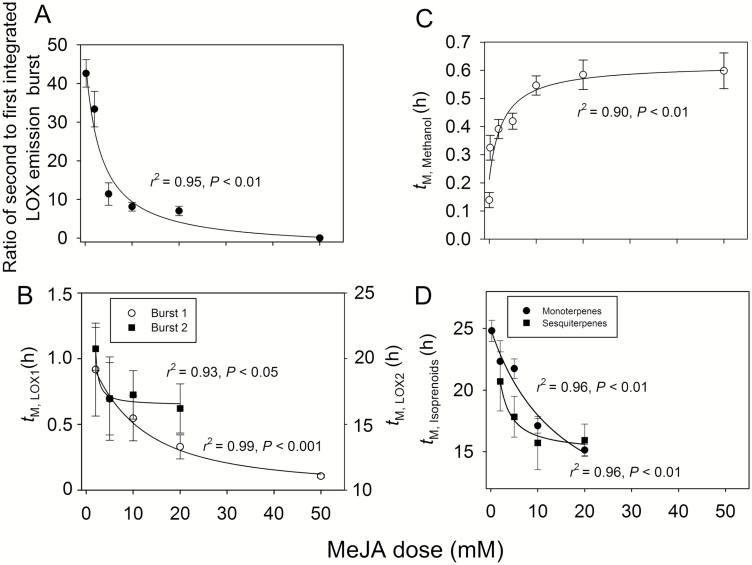
The ratio of the second to the first integrated LOX emission burst (*I*_TLOX2_/*I*_TLOX2_, A), and the times to the maxima of the first (*t*_M,LOX1_) and second (*t*_M,LOX2_) LOX emission bursts (B), emission burst of methanol (C) and the times to the maxima of monoterpene (*t*_M,monoterpene_) and sesquiterpene (*t*_M,sesquiterpene_) emission bursts (D) in relation to MeJA dose in leaves of *C. sativus* (see Fig. 2 for the sample responses and definition of the characteristics). MeJA treatments are as in Fig. 1 and data coverage is as in Fig. 3. In all cases, the data were fitted by hyperbolic regressions. For *I*_TLOX2_/*I*_TLOX2_ in (A), *y*=162/(3.27+*x*)–2.83, *r*^2^=0.95, *P*<0.01; for *t*_M,LOX1_ in (B), *y*=14.1/(11.7+*x*)–0.12, *r*^2^=0.99, *P*<0.001; for *t*_M,LOX2_ in (B), *y*=9.69/(0.0027+*x*)+15.7, *r*^2^=0.93, *P*<0.01; for *t*_M,methanol_ in (C), *y*=0.41*x*/(2.90+*x*)+0.21; for *t*_M,monoterpene_ in (D), *y*=281/(15.7+*x*)+7.03, *r*^2^=0.96, *P*<0.01; for *t*_M,sesquiterpene_ in (D), *y*=14.6/(0.50+*x*)+14.9, *r*^2^=0.96, *P*<0.01. Three replicate treatments at each MeJA application concentration were carried out.

The total integral emissions (Equation 1; Fig. 2 for definition) also scaled positively with the MeJA treatment concentration for both the faster (Fig. 4A, B) and the slower (Fig. 4A, C, D) emission responses. However, although the emission maxima at a given MeJA concentration were similar for the first and the second emission bursts of LOX compounds (Fig. 3A), the total LOX emission corresponding to the second emission burst was much larger than that for the first emission burst (Fig. 4A), and thus the total release of LOX compounds during the whole experiment mainly scaled with the slower LOX response (Fig. 4A). Stronger elicitation of the slower emission response was particularly evident for lower concentrations of MeJA such that the ratio of the second (*I*_T,LOX2_) to the first (*I*_TLOX,1_) integrated emission scaled negatively with MeJA concentration (Fig. 5A).

For total integrated emission versus MeJA concentration relationships, the non-linearity was much less than for maximum emission versus MeJA relationships (cf. Figs 3 and 4). In fact, the total LOX emissions corresponding to both the first and the second emission bursts were best fitted by linear regressions (Fig. 4A). Analogously with the maximum emissions, total LOX emissions were strongly correlated with the degree of leaf damage through the MeJA treatments (insets in Fig. 4).

### Timing and rate of elicitation of volatile emissions depend on MeJA concentration

MeJA concentration significantly affected the timing of volatile emission responses. The maxima for both the faster and the slower LOX emission bursts occurred earlier at higher MeJA concentration, whereas the change in the timing was greater for the faster LOX burst (Fig. 5B). In contrast, the maximum emission for the methanol burst occurred later at a greater MeJA concentration (Fig. 5C). Similarly to LOX, the maxima of monoterpene and sesquiterpene emissions also occurred earlier at higher MeJA concentrations, and this concentration response was stronger for monoterpenes (Fig. 5D).

**Fig. 6.**
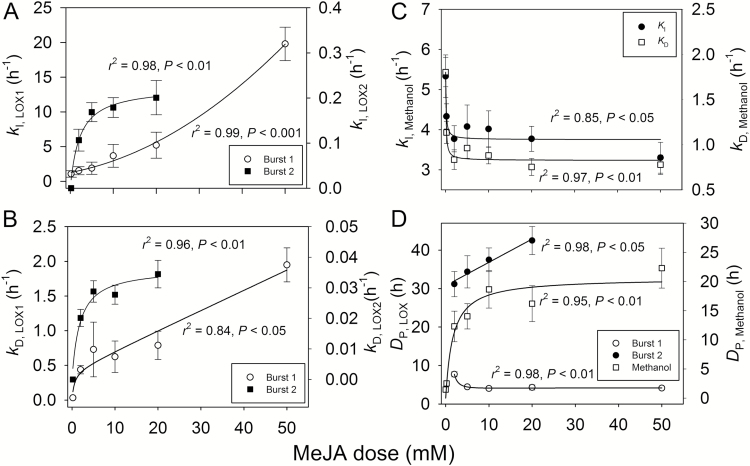
First-order rate constants (*k*_I_) for the initial increases (Equation 2, *k*_I_; A, C) and decreases (Equation 3, *k*_D_; B, C) of the MeJA-induced emissions for the first (*k*_I,LOX1_ and *k*_D,LOX1_) and the second (*k*_I,LOX2_ and *k*_D,LOX2_) emission burst of LOX volatiles (A, B) and methanol (C), and the duration (*D*_P_) of the first and second LOX compound emission burst and methanol emission burst (D) as dependent on the MeJA concentration in *C. sativus* leaves (see Fig. 2 for sample responses). Experimental treatments are as in Fig. 1 and data coverage is as in Fig. 3. The regressions describing the statistical effects of MeJA on the rate constants were the following: *y*=0.0050*x*^2^+0.12*x*+1.27 for *k*_I,LOX1_ and *y*=0.214*x*/(1.87+*x*) for *k*_I,LOX2_ in (A), *y*=2.51(1–e^−0.028*x*^) for *k*_D,LOX1_ and *y*=0.0357*x*/(1.42+*x*) for *k*_D,LOX2_ in (B), *y*=3.75 + 0.194/(0.123+*x*) for *k*_I,methanol_ and *y*=0.826 + 0.0920/(0.094+*x*) for *k*_D,methanol_ in (C), *y*=4.24–3.11/*x*+20.1/*x*^2^ for *D*_P,LOX1_, *y*=30.8 + 0.604*x* for *D*_P,LOX2_, and *y*=20.6*x*/(1.65+*x*) for *D*_P,methanol_ in (D). Three replicate treatments at each MeJA application concentration were carried out.

**Fig. 7.**
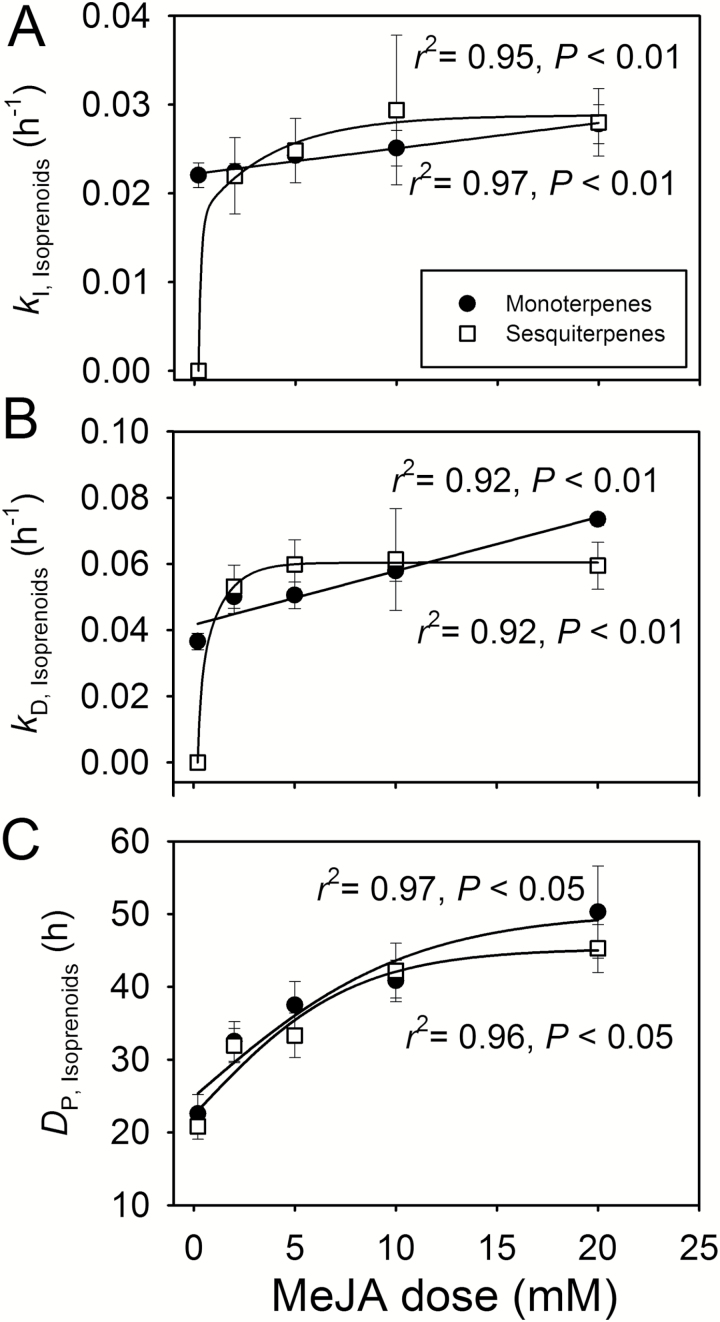
First-order rate constants (*k*_I_) for the initial increases (Equation 2, *k*_I_; A) and decreases (Equation 3, *k*_D_; B) of the MeJA-induced emissions for mono- (*k*_I,monoterpene_ and *k*_D,monoterpene_) and sesquiterpenes (*k*_I,sesquiterpene_ and *k*_D,sesquiterpene_) and the durations (C) of monoterpene (*D*_P,monoterpene_) and sesquiterpene (*D*_P,sesquiterpene_) emission bursts in relation to MeJA concentration in *C. sativus* leaves (see Fig. 2 for sample responses). Experimental treatments are as in Fig. 1 and data availability is as in Fig. 3. The regressions fitted to the data as: *y*=0.0029*x*+0.22 for *k*_I,monoterpene_ and *y*=0.297*x*/(0.736+*x*) for *k*_I,sesquiterpene_ in (A), *y*=0.0016*x*+0.041 for *k*_D,monoterpene_ and *y*=0.061*x*/(0.295+*x*) for *k*_D,sesquiterpene_ in (B), *y*=50.3/[1+e^(0.107−*x*)/5.26^] for *D*_P,monoterpene_ and *y*=45.3/[1+e^(0.0784−*x*)/3.84^)] for*D*_P,sesquiterpene_ in (C). Altogether three replicate treatments at each MeJA application concentration were carried out.

To characterize further the MeJA effects on the emission kinetics, the induction (*k*_I_, Equation 2) and decay (*k*_D_, Equations 2 and 3) rate constants (see also Table 1) were determined. This analysis indicated that both the initial increase and decrease of emissions was faster at higher MeJA concentrations for LOX compounds (Fig. 6A, B) and terpenoids (Fig. 7A, B). However, for methanol, the rate constants *k*_I_ and *k*_D_ initially decreased over the MeJA concentration range of 0.2–5 mM, and they were weakly affected by MeJA concentrations >5 mM (Fig. 6C). Both the rise and decline kinetics were much faster for the first than for the second LOX emission burst (Fig. 6A, B). In addition, MeJA concentration dependencies of *k*_I_ and *k*_D_ were weaker for the second LOX emission burst, with a moderate change in the induction and decay rates over the MeJA concentration range of 5–20 mM (Fig. 6A, B). The induction and decay rates were similar for mono- and sesquiterpenes, except for the lowest MeJA treatment concentration of 0.2 mM that barely induced sesquiterpene release (Fig. 6C, D).

Despite a faster rate of decline at greater MeJA concentrations, induction to a higher maximum level at greater MeJA concentration (Fig. 3) implied that the total length of the emission burst increased with increasing MeJA concentration for all compound classes (Figs 6D, 7C), except for the first LOX burst (Fig. 6D). In the case of the early LOX release, the duration of the pulse length decreased between 2mM and 5 mM MeJA, and was thereafter invariable (Fig. 6D).

## Discussion

### Exposure to MeJA leads to rapid bursts of characteristic stress volatiles in *Cucumis sativus*

A variety of biotic stresses elicits emissions of volatile products of the lipoxygenase pathway (LOX compounds, also called green leaf volatiles) (Matsui *et al.*, 2012; Scala *et al.*, 2013). LOX compounds are synthesized by multiple lipoxygenases and fatty acid hydroperoxide lyases, several of which are constitutively active in leaves (Feussner and Wasternack, 2002), implying that as soon as the substrates, polyunsaturated fatty acids, are released from membranes, LOX products are rapidly emitted. Typically, the early LOX response occurs within 2–30 min after biotic stress treatment (Zhang and Xing, 2008; Bruinsma *et al.*, 2009; Brilli *et al.*, 2011; War *et al.*, 2012; Portillo-Estrada *et al.*, 2015), and this rapid LOX burst has been shown to be involved in priming and triggering subsequent local and systemic stress responses (Farag and Paré, 2002; Mithöfer *et al.*, 2005; Niinemets *et al.*, 2013; Scala *et al.*, 2013).

In our study, LOX emissions were detected immediately after enclosure of treated leaves in the measurement system, and the emissions reached the first maximum in 0.1–1 h depending on MeJA concentration (Figs 2A, 5B). The increase was faster than the decrease (cf. Fig. 6A, B) such that the emissions corresponding to this initial burst were maintained at a moderately high level for several hours after the MeJA treatment (Fig. 2A). Thus, this early MeJA response clearly reflects activation of constitutive lipoxygenases (LOX), hydroperoxide lyase (HPL), allene oxide synthase (AOS), and alcohol dehydrogenase (ADH), resembling the rapid response to wounding herbivores (Brilli *et al.*, 2011; Portillo-Estrada *et al.*, 2015).

Concomitant with the start of LOX emissions, major methanol emissions were elicited (Fig. 2D). Release of methanol is a characteristic stress response that reflects activation of demethylation of cell wall pectins by constitutively expressed pectin methylesterases (Micheli, 2001; Pelloux *et al.*, 2007). Previous studies have demonstrated activation of methanol release upon leaf mechanical wounding (Brilli *et al.*, 2011; Portillo-Estrada *et al.*, 2015), insect feeding (Peñuelas *et al.*, 2005; von Dahl *et al.*, 2006), fungal infestation (Copolovici *et al.*, 2014*b*; Jiang *et al.*, 2016*b*), and ozone exposure (Beauchamp *et al.*, 2005). Such cell wall modifications can importantly enhance the penetration of downstream signaling compounds (Gális *et al.*, 2009), including MeJA diffusion. Furthermore, there is evidence that in addition to LOX volatiles, methanol is not only the by-product of stress-dependent cell wall modifications, but also can serve as an important signal eliciting or modifying systemic stress responses (von Dahl *et al.*, 2006; Seco *et al.*, 2011; Hann *et al.*, 2014; Komarova *et al.*, 2014).

### Biphasic MeJA elicitation of volatile emissions in *C. sativus*

The fast emission burst of LOX and methanol was followed by slower emission bursts of LOX (Fig. 2B), monoterpenes (Fig. 2C), and sesquiterpenes that reached a maximum in 16–25 h after MeJA treatment (Fig. 5B, C). Constitutive monoterpene emissions are very low in non-stressed cucumber (Table 2), and the emissions were not significantly elicited by MeJA over the short term, suggesting that the longer term elicitation of terpenoid emissions reflects a gene expression level response as has been observed in several studies looking at MeJA-dependent stress responses (Martin *et al.*, 2002, 2003; Byun-McKay *et al.*, 2006). Furthermore, the induced monoterpene blend significantly differed from the blend of constitutive emissions (Table 2), further supporting the argument that the induced monoterpene emissions reflected expression of inducible terpenoid synthases not expressed under non-stressed conditions. The terpenoids elicited by MeJA in our study (Table 2), especially monoterpenes (limonene and linalool) and sesquiterpenes (β-farnesene, α-cedrene, and β-caryophyllene), are also induced in herbivore-infested cucumber (Bouwmeester *et al.*, 1999; Kappers *et al.*, 2010), and play key roles as airborne signals attracting herbivore enemies or in priming defenses in neighboring plants (Degenhardt and Lincoln, 2006; Brilli *et al.*, 2009; Mäntylä *et al.*, 2014).

As previous studies have demonstrated, biosynthesis of terpenes is delayed after the initiation of biotic stress (Arimura *et al.*, 2008, 2009), reflecting the lags in signal transduction as well as the fact that the transcription and translation of terpene synthases are time-consuming. However, the existence of a second sustained burst of LOX compounds synchronously with the elicitation of induced terpene emissions after ~10 h is surprising. Because it occurred simultaneously with terpenoid emissions, this second burst of LOX compounds is unlikely to be the chemical elicitor inducing terpenoid release. In fact, there is evidence that the late MeJA response reflects the rise of endogenously synthesized jasmonate levels (Tamogami *et al.*, 2008), and, thus, the second release of LOX might indicate the onset of jasmonate-dependent gene expression as reported in several previous studies for lipoxygenase pathway genes (Bell and Mullet, 1991; Wasternack and Parthier, 2007).

From a biological perspective, LOX compounds are not only released upon immediate wounding in herbivore-damaged leaves, but also concomitantly with the synthesis of terpenoids elicited at the later stages of induction. Moreover, these LOX-containing compound blends play important roles in attraction of herbivore enemies and in priming of neighboring plants (Dicke *et al.*, 1999; Bruinsma *et al.*, 2007; Frost *et al.*, 2008; Allmann and Baldwin, 2010; Copolovici *et al.*, 2011, 2014*a*). Although the exposure to MeJA does not fully mimic the herbivory stress (Dicke *et al.*, 1999; Degenhardt and Lincoln, 2006; Kappers *et al.*, 2010), it still elicits a blend of volatiles that attracts enemies of herbivores similarly to genuine herbivore feeding (Dicke *et al.*, 1999; Kappers *et al.*, 2010). Thus, this second LOX burst concomitant with the induced terpenoid emissions might be part of the characteristic herbivory smell bouquet that is driving the plant–insect and plant–plant interactions.

Although longer term kinetic studies are rare, especially those considering the entire bouquet of volatiles, biphasic emission kinetics have been demonstrated in response to various stresses for several volatiles, including biphasic methanol emissions upon ozone stress (Beauchamp *et al.*, 2005), biphasic ethylene emissions in response to treatments with the fungal elicitor cryptogein and infections by *Phytophtora parasitica* (Wi *et al.*, 2012) and *Pseudomonas syringae* (Mur *et al.*, 2008), and biphasic LOX product and terpenoid emissions in response to infections by *Melampsora epitea* (Toome *et al.*, 2010). However, with the exception of the ozone stress study of Beauchamp *et al.* (2005) that was carried out with a proton-transfer reaction quadrupole mass spectrometer (PTR-QMS), other kinetic studies had much lower time resolution than that (10 s) used in our study. While the elicitation of the volatile response to mechanical wounding and herbivory is characteristically very fast (Brilli *et al.*, 2011; Copolovici *et al.*, 2011, 2014*a*; Portillo-Estrada *et al.*, 2015), resembling the MeJA treatment response in our study, both the first and the second emission bursts in the pathogen-infected leaves (Mur *et al.*, 2008; Toome *et al.*, 2010; Wi *et al.*, 2012) occurred later than in our study in MeJA-treated leaves (Fig. 2A, D). Clearly, different stresses can propagate differently, reflecting the diversity of biological characteristics of impacting organisms as well as differences in stress perception and signal transduction. Further research using high-resolution techniques as used here is needed to resolve the general and specific features of shorter and longer term elicitation responses induced by different stresses, including studies linking the emissions to pertinent gene expression patterns.

### Emission rates and total emission of induced volatiles scale with MeJA dose

The level of biological stress varies greatly depending on the degree of infestation by herbivores and infection by pathogens (Niinemets *et al.*, 2013), but biological stress severity versus stress response relationships have not been routinely studied. Here we observed that MeJA treatment concentration and the degree of leaf area damaged were quantitatively correlated (Fig. 1), in agreement with past observations indicating that exogenous application of MeJA can result in local phytotoxicity (Heijari *et al.*, 2005). We also observed that both the total and maximum emissions of LOX compounds for both the early and the second emission burst (Figs 3A, 4A), for the early methanol emission burst (Figs 3B, 4B), and for the late monoterpene (Figs 3C, 4C) and sesquiterpene (Figs 3D, 4D) emission bursts were quantitatively associated with MeJA concentration and the proportion of leaf area damaged (damage severity). Thus, these results indicate that both the initial stress response due to activation of constitutive defenses and the later response due to activation of induced defenses are dose dependent.

MeJA that mainly enters through the stomata, and to a lower degree through the cuticle, is expected to become progressively diluted as it penetrates deeper into the leaf interior and dissolves in leaf water. Thus, the dose dependence of the early constitutive response as evident in the first LOX compound burst (Fig. 2A) and in the methanol burst (Fig. 2D) can result from quantitative scaling of the proportion of impacted cell wall and membrane sites with MeJA concentration. Such a positive scaling of early LOX and methanol emission responses with the degree of impact has been observed in wounding experiments (Brilli *et al.*, 2011; Portillo-Estrada *et al.*, 2015), and suggests that the control of the rapid elicitation response at the level of immediate impact is possible.

In the case of the late-induced response, the situation is less clear because in addition to MeJA *per se*, the primary MeJA-induced LOX volatile emissions can propagate the signal to cellular locations not necessarily directly impacted by MeJA (Cardoza *et al.*, 2002; Engelberth *et al.*, 2007; Kishimoto *et al.*, 2008; Piesik *et al.*, 2013; Castelyn *et al.*. 2015).

The MeJA dose dependence of induced emissions resembles herbivory experiments where the rate of emissions of mono- and sesquiterpenes increases with the proportion of leaf area consumed (Copolovici *et al.*, 2011, 2014*a*), indicating that the rate of terpene synthesis becomes progressively greater in the remaining tissues. Analogously, in fungal-infected leaves, terpenoid emissions increase with increasing spread of the necrotic area (Jiang *et al.*, 2016*b*). We argue that the quantitative relationships between the dose of the model compound MeJA, severity of damage, and the volatile emission responses in cucumber have major biological consequences. Studies have demonstrated that different levels of induction of volatiles by MeJA treatment alter both plant attractiveness to herbivores (Heil, 2014) and the repellency to herbivores (Zas *et al.*, 2011). This suggests that the capacity to respond to a biotic stress in a dose-dependent manner as demonstrated here provides significant fitness advantages.

### A conceptual model describing MeJA elicitation of defenses from stress response to acclimation

The induction of volatiles released upon MeJA treatment from cucumber leaves followed similar elicitation patterns for both the fast and slow phases, with an initial exponential or sigmoidal increase to a maximum level, followed by a decrease to the baseline emission (Fig. 2). We fitted the temporal shapes of the increase and decrease of volatiles by first-order exponential relationships to characterize the rise and decay kinetics (Equations 2 and 3) and further characterized the timing of emission elicitation (Fig. 2; Table 1). Both the rate of increase and the decrease of LOX compounds and terpenoids were strongly enhanced by increasing MeJA concentrations (Figs 6A, B, 7A, B). Furthermore, both the first and the second LOX burst (Fig. 5B) and monoterpene and sesquiterpene emission bursts (Fig. 5D) occurred earlier, and were sustained for longer time periods (Figs 6D, 7C) at higher MeJA dose. This evidence emphasizes the highly dynamic nature of the MeJA concentration dependence of volatile emissions over both the short and long term.

**Fig. 8.**
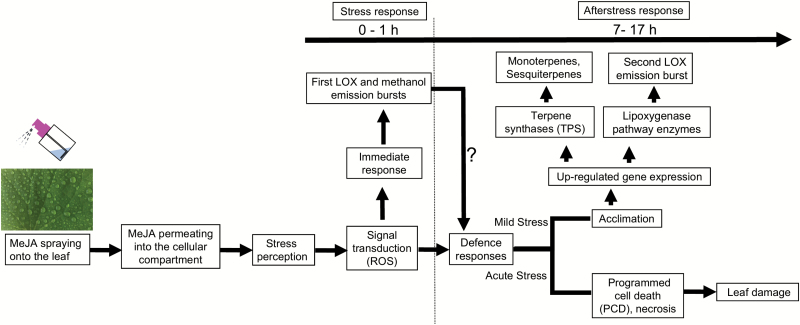
A conceptual model of elicitation of short- and long-term volatile responses upon MeJA treatment of cucumber leaves. Gaseous MeJA is taken up into the leaf internal air space through stomata, permeates further through cell walls and plasmalemma, reaching ultimately the symplastic leaf compartments. This activates an oxidative burst due to rapid formation of reactive oxygen species (ROS; Garrido *et al.*, 2003; Zhang and Xing, 2008; Küpper *et al.*, 2009; Hazra *et al.*, 2017) and release of free polyunsaturated fatty acids from plant membranes (Dicke *et al.*, 1999; Feussner and Wasternack, 2002; Andreou and Feussner, 2009) in several minutes after the treatment at the immediate location of MeJA impact. Due to the constitutive activity of lipoxygenases, free fatty acids are rapidly converted to volatile lipoxygenase pathway products (LOX products; Feussner and Wasternack, 2002; Andreou and Feussner, 2009), resulting in the first burst of LOX emissions (Fig. 2A). Simultaneously with the release of free fatty acids or maybe even somewhat earlier, constitutive pectin methylesterases (Micheli, 2001; Pelloux *et al.*, 2007) are activated, resulting in major emissions of methanol (Fig. 2D). In a longer-term sequence of events probably involving endogenous jasmonate formation and interplay with jasmonate repressor proteins (JAZ) (Chini *et al.*, 2007; Wasternack and Song, 2017; Zhang *et al.*, 2017), plant defense responses depend on the initial stress severity. Mild and moderate stress caused by MeJA treatment (5, 10, and 20 mM) is expected to lead to elicitation of gene expression level defenses including enhanced expression of terpenoid synthase (TPS; Martin *et al.*, 2002, 2003; Byun-McKay *et al.*, 2006) and lipoxygenase pathway genes (Bell and Mullet, 1991; Wasternack and Parthier, 2007), resulting in volatile terpene and LOX emissions that are sustained for long time periods of ~20–30 h (Fig. 2B, C). In the case of moderate stress, propagation of necrotic lesions remains localized (Fig. 1A). In contrast, acute MeJA stress (50 mM) leads to enhanced progression of programmed cell death (PCD), and subsequent rapid propagation of necrosis over the entire leaf surface (Fig. 1A).

For chronic biotic stresses such as herbivory infestation and pathogen attacks, quantitative relationships between the severity of biotic stress and release of induced volatiles have been suggested to result from the scaling of emissions with propagation of damage and the number of simultaneous stress impact sites (Niinemets *et al.*, 2013). As this study with MeJA elicitor demonstrates, the scaling relationships of volatile responses with MeJA dose, the ‘stress severity’, consist of both local and systemic responses. As for the local early response, it indeed can reflect scaling of emissions with the spread of the immediate stress impact sites (Fig. 8). However, the quantitative scaling of the subsequent slower gene-level responses with MeJA dose is obviously more complex and probably reflects a systemic response. The timing and magnitude of this systemic response is determined by the initial MeJA dose, but the way in which the early stress signal determines the gene expression response is still unclear at a mechanistic level, and will require further studies looking at both the activation of expression of regulator and target genes. It is likely that the balance between the free transcription regulator MYC2 that activates downstream jasmonate transcriptional responses and jasmonate signaling repressor proteins (JAZ proteins) that negatively affect MYC2 levels (Gális *et al.*, 2009), together with the elicitation of endogenous jasmonate synthesis (Tamogami *et al.*, 2008), determines the onset of elicitation of gene expression and subsequent repression.

Furthermore, the set of events downstream of the initial MeJA impact consists of acclimation responses and localized acute necrotic or PCD-like responses with the share among them determined by the initial MeJA concentration (Figs 1, 8). Thus, with increasing MeJA concentration, a greater proportion of leaf area undergoes death, while the volatile emission capacity of the remaining cells is increasingly up-regulated. Stronger amplification of the emission capacity in those cells still alive resembles the emission response to herbivory where foliar terpenoid emissions in remaining leaf parts increase dependent on the proportion of leaf area consumed by herbivores (Copolovici *et al.*, 2011, 2014*a*, 2017).

What could be the biological significance of the dose dependence of the second elicited emission burst? Due to high reactivity in the ambient atmosphere, the volatile signal itself fades with the distance from the emission source (Holopainen and Blande, 2013; Holopainen *et al.*, 2013; Blande *et al.*, 2014). Thus, a stronger signal will reach more distant leaves, and also provides a farther reaching signal for other organisms such as herbivore predators. Furthermore, we suggest that the strength of the volatile signal might carry information about the severity of the biological impact and, as such, contribute to stronger priming responses in surrounding leaves of the same plant and neighboring plants. We argue that for quantitative prediction of biotic stress severity versus emission response relationships, further studies are needed to gain insight into the turnover of JAZ and MYC2 proteins and into the timing and magnitude of formation of endogenous jasmonate as driven by the severity of biotic stress impact.

### Conclusions

High time resolution measurements conducted here have highlighted the biphasic kinetics of volatile emissions induced by MeJA treatment. Our study demonstrates rapid constitutive lipoxygenase pathway volatile (LOX) and methanol emissions and subsequent elicitation of terpenoid emission. Strong quantitative relationships between the timing and magnitude of early and late emissions and applied MeJA concentration collectively indicate high plant plastic capacity to respond to biotic stress, and emphasize the highly dynamic nature of the MeJA concentration dependence of volatile emissions over both the short and long term. Albeit that this study presented exciting evidence of quantitative scaling of local and systemic emission responses to MeJA treatment, we suggest that to gain insight into the mechanisms of regulation of the magnitude and kinetics of the downstream responses and validate the differences in the sensitivity of gene expression in cucumber, further studies should look at expression of terpenoid synthase genes under treatments with different MeJA concentrations. In addition, higher resolution reactive oxygen species measurement techniques should be developed to obtain complementary information on leaf oxidative status through the emissions bursts recorded by PTR-TOF-MS.
